# Measuring Coverage in MNCH: Total Survey Error and the Interpretation of Intervention Coverage Estimates from Household Surveys

**DOI:** 10.1371/journal.pmed.1001386

**Published:** 2013-05-07

**Authors:** Thomas P. Eisele, Dale A. Rhoda, Felicity T. Cutts, Joseph Keating, Ruilin Ren, Aluisio J. D. Barros, Fred Arnold

**Affiliations:** 1 Department of Global Health Systems and Development, Tulane University School of Public Health and Tropical Medicine, New Orleans, Louisiana, United States of America; 2 Center for Analytics and Public Health, Battelle Memorial Institute, Columbus, Ohio, United States of America; 3 Independent consultant, La Londe les Maures, France; 4 Demographic and Health Surveys, ICF International, Calverton, Maryland, United States of America; 5 Federal University of Pelotas, Pelotas, Brazil; Professor of Demography and Social Statistics, University of Southampton, United Kingdom

## Abstract

Nationally representative household surveys are increasingly relied upon to measure maternal, newborn, and child health (MNCH) intervention coverage at the population level in low- and middle-income countries. Surveys are the best tool we have for this purpose and are central to national and global decision making. However, all survey point estimates have a certain level of error (total survey error) comprising sampling and non-sampling error, both of which must be considered when interpreting survey results for decision making. In this review, we discuss the importance of considering these errors when interpreting MNCH intervention coverage estimates derived from household surveys, using relevant examples from national surveys to provide context. Sampling error is usually thought of as the precision of a point estimate and is represented by 95% confidence intervals, which are measurable. Confidence intervals can inform judgments about whether estimated parameters are likely to be different from the real value of a parameter. We recommend, therefore, that confidence intervals for key coverage indicators should always be provided in survey reports. By contrast, the direction and magnitude of non-sampling error is almost always unmeasurable, and therefore unknown. Information error and bias are the most common sources of non-sampling error in household survey estimates and we recommend that they should always be carefully considered when interpreting MNCH intervention coverage based on survey data. Overall, we recommend that future research on measuring MNCH intervention coverage should focus on refining and improving survey-based coverage estimates to develop a better understanding of how results should be interpreted and used.

## Abstract 2

In a *PLOS Medicine* Review, Thomas Eisele and colleagues discuss the importance of considering sampling and non-sampling errors when interpreting estimates of coverage of maternal, newborn, and child health interventions based on data from household surveys.

*This paper is part of the* PLOS Medicine “*Measuring Coverage in MNCH” Collection*

## Introduction

Nationally representative household surveys are increasingly relied upon to measure maternal, newborn, and child health (MNCH) intervention coverage at the population level in low- and middle-income countries. These surveys include the Demographic and Health Survey/s (DHS), the Multiple Indicator Cluster Survey/s (MICS), the AIDS Indicator Survey/s (AIS), and the Malaria Indicator Survey/s (MIS). These surveys rely on scientific sampling methods, which require each element of the target population to have a known and non-zero probability of selection, to obtain point estimates of MNCH intervention coverage at the national and sub-national levels every 3–5 years [1]. Because accurate up-to-date sampling frames of individuals and households are often unavailable in many low- and middle-income countries, these surveys typically use a multi-stage cluster sample design. Clusters (primary sampling units) are selected at the first stage with a probability proportional to size strategy, with size being the estimated population size of the cluster. A constant number of households is then randomly selected at the second stage of selection from a sampling frame of households created from a complete enumeration of households in the selected clusters. Household surveys have become increasingly standardized in their sampling approaches and questionnaire designs to produce comparable results across countries and over time.

In using the coverage figures for MNCH interventions from household surveys for programmatic and policy decisions, it is important to remember that surveys only provide estimates of the true population characteristics of interest. No matter how large a sample is drawn, and no matter how well a survey is designed or implemented, all survey point estimates have a certain level of error (total survey error) comprising sampling and non-sampling error, both of which need to be considered when interpreting survey results. Sampling error—the difference between the sample estimate of a population characteristic and the real value of the population characteristic—is the result of sampling from the population rather than taking measurements from the entire population [2]. Non-sampling error includes all other sources of error. For multi-stage cluster sampling of households in low- and middle-income countries, the most common types of non-sampling error are information bias and selection bias but coverage bias and non-response bias are also important issues.

In this review, which is part of the *PLOS Medicine* “Measuring Coverage in MNCH” Collection, we discuss the important sampling error and non-sampling error issues involved in interpreting MNCH intervention coverage estimates derived from household surveys for decision making using relevant examples from national surveys to provide context.

## Sampling Error and Its Implications

### Confidence Intervals

We most often think of sampling error as the precision of a point estimate, represented by 95% confidence intervals. Confidence intervals are useful for two purposes. First, they characterize the precision of the estimate. Second, they provide context about whether estimated parameters are likely to be equal between two populations or time-points. In the absence of non-sampling error, if a large number of repeated samples is taken from the target population using the same sampling design, 95% of the resultant 95% confidence intervals about each sample point estimate will contain the true, albeit unknown, intervention coverage in the population [3]. In practice, the interpretation of confidence intervals is slightly different. Suppose, for example, we have a point estimate of intervention coverage of 70%, with a 95% confidence interval of 65%–75% in a program for improving MNCH. This confidence interval is typically taken to mean that we are 95% certain that the true intervention coverage in the population lies somewhere between 65% and 75%. Consider if the program had set a target of achieving 70% population coverage. In this case, one could not say with a high degree of confidence that the program had achieved its target coverage, even with a point estimate for coverage at 70% since fully half of the confidence limit lies below 70%.

### Sample Size and Sampling Design

The level of sampling error around a point estimate depends on the number of observations selected from the target population (sample size), the underlying prevalence and variance of the characteristic of interest in the target population, and the sampling design used in selecting the sample. In general, for a given sampling design, the precision of a point estimate improves with increasing sample size. Thus, a larger sample size yields a tighter confidence interval. However, given a specified sample size, the sampling design also directly affects the precision of an intervention coverage point estimate. In practice, data from nationally representative surveys nearly always come from a cluster survey rather than a simple random sample of households. In cluster surveys, the sampling error is affected by the number of respondents per group as well as the intracluster (or intraclass) correlation coefficient, which provides a measure of how similar the elements being analyzed in each cluster are with respect to a particular characteristic of interest [2]. In general, for a given sample size, the precision of a point estimate ascertained from a cluster sample design decreases as the sample size per cluster increases, and as the intracluster correlation coefficient increases.

### Reporting Sampling Errors and Confidence Intervals

Although the sample size and sampling design are frequently reported in survey methods, sampling errors represented by confidence intervals are rarely reported in the main body of reports of national surveys. DHS survey reports and reports for AIS and MIS surveys conducted through the DHS program include standard errors and accompanying confidence intervals of ±2 standard errors (slightly wider than the 95% confidence interval) for most key indicators in Appendix B only. In MICS survey reports, standard errors and 95% confidence intervals appear in Appendix S. Almost no MIS surveys conducted outside of the DHS program include sampling errors in the survey reports or appendices.

### Comparing Survey Results

**Figure 1:**
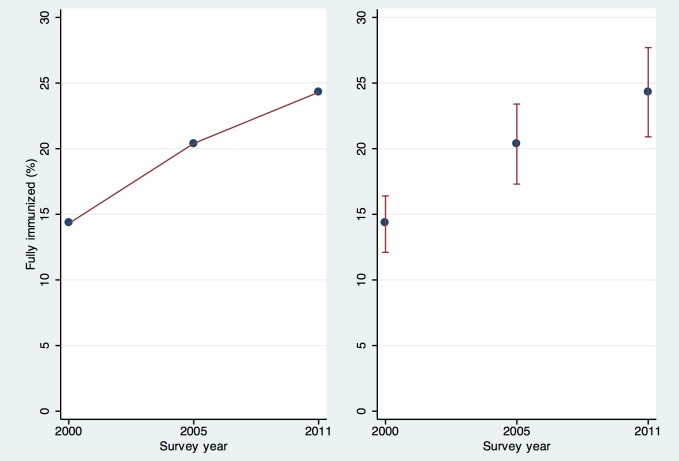
Two ways of looking at coverage of full immunization in Ethiopia—with and without confidence intervals. Ethiopia DHS surveys 2000, 2005, 2011 [33].

One oft-cited advantage of large well-conducted surveys is that their results can be compared meaningfully both over time and between locations. In the absence of a formal hypothesis test, informal conclusions about the statistical significance of a difference are sometimes drawn by examining whether two confidence intervals overlap. Indeed, if two 95% confidence intervals do not overlap, then a formal hypothesis test would reject the null hypothesis of equality with a *p*-value below 5%. Unfortunately the converse is not true. If 95% confidence intervals overlap, it is still possible for the *p*-value from the hypothesis test to fall below 5% [4],[5]. An example from the 2011 Ethiopia DHS report (presented in Box 1 and Figure 1) illustrates how difficult it can be to correctly interpret MNCH intervention coverage estimates without considering confidence intervals [6].

**Box 1. An Illustration of the Difficulty in Correctly Interpreting MNCH Intervention Coverage Estimates without Considering Confidence Intervals**

The 2011 Ethiopia DHS survey report states “The percentage of children age 12–23 months who were fully vaccinated at the time of the survey increased from 14% in 2000 to 20% in 2005 and 24% in 2011—a 70% increase over 10 years and a 19% increase in the 5 years preceding the 2011 survey” [6]. The main text of the Ethiopia report does not present confidence intervals, but Appendix B shows that for the 2011 survey the approximate 95% confidence interval for the immunization coverage of 24% was 21%–28%. In the 2005 survey, the confidence interval around the point estimate of 20% was 17%–23%, and in 2000 the confidence interval around the point estimate of 14% was 12%–16% [33]. Although a statistical test for significance would be needed to draw a strong conclusion, the difference between 2005 and 2011 (20% up to 24%) is not likely to be statistically significant at the 5% level because the confidence intervals overlap (see Figure 1). It would have been helpful for the body of the report to note the *p*-value associated with the 19% increase, or to say that the 2005 to 2011 increase is not statistically significant at the 5% level. This example demonstrates the importance of considering confidence intervals when survey-based estimates are used to draw strong conclusions or to make important programmatic decisions.

### Sampling Error and Data Disaggregation

When analyzing survey data for MNCH coverage indicators, sampling errors and the accompanying confidence intervals are influenced, at times substantially, by changes in sample size owing to disaggregating the data by socio-behavioural or demographic characteristics of interest, such as where the respondents live or their household socioeconomic status. While DHS and MICS surveys typically report the confidence intervals around many key indicators in their appendices, they do not report confidence intervals of disaggregated point estimates. To illustrate why it is important to consider the sampling error of such estimates, we present summary sampling characteristics of established malaria control coverage indicators from the 2007 Zambia DHS survey (Table 1) [7]. Point estimates were weighted to account for unequal probability sampling and any differential non-response. Standard errors were estimated to account for correlated data at the primary sampling unit level using the Huber White Sandwich estimator, which accounts for the loss of precision due to increased intracluster correlation coefficient.

**Table 1:** Sampling characteristics of selected point estimates from the 2007 Zambia DHS Survey [7].

Indicator	Percent Point Estimate	Percent Standard Error	95% CI	Sample Size
**Percent of households with ≥1 ITN**	52.2	1.24	49.8–54.7	7,164
**Percent of children <5 years old with fever in past 2 weeks**	17.7	0.71	16.4–19.2	5,844
**Percent of children <5 years old with fever who received any antimalarial**	38.2	1.85	34.6–41.9	1,034
Wealth quintile^a^				
Lowest	35.3	3.85	27.7–43.0	219
Second	42.6	3.60	35.5–49.7	228
Middle	36.6	3.44	29.8–43.4	253
Fourth	37.5	3.15	31.3–43.8	228
Highest	39.6	5.02	29.5–49.7	106

Point estimates in this table may vary slightly from the point estimates reported in the 2007 Zambia DHS survey because of slight differences in inclusion criteria during analysis, although all are within 1%.

a There are different numbers of children in each wealth quintile because wealth quintiles are calculated at the household level for all persons in the household and not for subgroups.

For assessing progress of malaria control efforts in Zambia, the standard indicator for household insecticide-treated mosquito net (ITN) coverage shows that 52% of households possess at least one ITN, with a 95% confidence interval of ±2.5 percentage points (Table 1). The interpretation for programmatic purposes, based on information from the sample of 7,164 households, would be that one has 95% confidence that the true (albeit unknown) proportion of households in Zambia with at least one ITN lies somewhere between 50% and 55%, in the absence of non-sampling error. By contrast, the indicator used for assessing progress in the coverage of access to antimalarial treatment of fevers in children (38%) has a 95% confidence interval of ±3.8 percentage points, because this sample is limited to only those children with fever in the past 2 weeks (5,844 children). When disaggregated by household socioeconomic status, the 95% confidence intervals increase considerably. For example, based on a subsample of 106 children, the proportion with a fever in the past 2 weeks in the wealthiest households who received an antimalarial was estimated to be 40%, with a 95% confidence interval of ±10 percentage points, which would be interpreted to mean that one can only be 95% confident that the true antimalarial coverage lies somewhere between 30% and 50% in this population in Zambia. This level of uncertainty clearly poses challenges in the interpretation of the point estimate for decision making.

## Non-sampling Error and Its Implications

Unlike sampling error, the direction and magnitude of non-sampling error is almost always unmeasurable, and therefore unknown. Non-sampling error cannot be controlled directly by sample size or by type of probability sampling design used.

Non-sampling error is more insidious than sampling error and can develop at many stages of the survey. Study planners need to anticipate as many threats to the validity of the survey results as possible and put careful controls in place to limit the magnitude of non-sampling error, such as the strict use of probability sampling, sensitization to increase response rates, high quality training of data collectors, and implementation of quality control measures for field work. Another paper in this collection on vaccination coverage provides more details of sources of non-sampling error and measures to mitigate against them [8].

Importantly, although sampling error is generally reduced by increasing sample size, non-sampling error can have an inverse relationship with sample size. Together, these factors make interpreting MNCH intervention coverage estimates obtained from household surveys very challenging in the presence of substantial non-sampling error. Non-sampling errors are also a major concern when comparing survey results from different surveys using different survey instruments or survey methodology, or inadequate levels of quality control. Below we present examples of the more common types of non-sampling errors and biases that threaten MNCH intervention coverage estimated from household surveys.

### Information Error and Information Bias

Information error and information bias (also referred to as measurement error and bias) are common in household survey estimates of MNCH intervention [8]–[13]. Information error arises from errors in measuring MNCH intervention coverage, which often occur when the respondents do not know the exact answer to the survey questions, yet provide answers anyway. Information bias arises from systematic (i.e., non-random) errors in measuring MNCH intervention coverage and includes recall bias and social-desirability bias. Although information error typically results in higher variance, and as a result decreased precision of the point estimate, information bias results in an overestimate or underestimate of the population point estimate. The real problem is that one does not know which way this type of error is biasing the results.

The measurement of vaccination coverage provides a good example of how information error and bias can affect the ability of decision makers to use information from surveys to evaluate the trends in coverage over time. As described by Cutts and colleagues elsewhere in this collection [8], information on vaccination coverage is usually kept in home-based vaccination records but once a child is past infancy, parents may not retain these records. If a vaccination card is not available, DHS and MICS surveys request information from each mother on the vaccinations received by children born in the past 5 years. Because home-based vaccination records are less likely to be available for children born 4 years ago than for those born 1 year ago, recall error or bias may be more likely for older than for younger children, which reduces the ability of decision makers to draw strong conclusions about trends.

Even with the best designed surveys and questionnaires, recall error and bias can affect MNCH intervention coverage estimates [14]–[16]. For example, for estimating ITN coverage, a key indicator for malaria control programs [17], surveys must ascertain the type of net used and, for nets that are not long-lasting insecticidal nets, when the net was procured and treated with insecticide to distinguish nets that are ITNs from those that are not. Data from Eritrea show substantial date heaping (rounding off of time since an event by respondents) at 12 months for both net procurement and insecticide re-treatment. Date heaping affects whether nets meet the definition of an ITN and therefore has the potential to bias coverage estimates [18].

Measurement of ITN use by children <5 years old also provides a good example of how social desirability bias can impact MNCH coverage estimates. Suppose a national survey obtained an estimate of ITN use among children of 60%, with a 95% confidence interval of 55%–65% but that respondents in selected households tended to tell household interviewers that their ITNs were being used more often than they really were because the respondents feel socially obligated to respond positively to questions about recent net use, a situation that is thought to occur [19]. In this scenario, social desirability bias results in systematic over-reporting of the use of ITNs. Even in a well-designed and implemented survey with a sampling error ±10%, we cannot be 95% certain that the true proportion of child ITN use falls within the calculated 95% confidence interval of 55%–65%. In fact, we would need to use expert but subjective judgment as to the direction and the magnitude of any bias when interpreting this coverage estimate and the degree to which bias may affect results.

### Selection Bias

Another common form of non-sampling error is selection bias, which occurs when potential respondents do not all have a known and non-zero probability of selection. In such a case, no matter how large a sample is taken, survey point estimates will always differ from the true, yet unknown, population characteristic under study [3]. Common sources of selection bias in surveys that measure MNCH intervention coverage include: (1) failure to include some eligible respondents in the sampling frame, (2) failure to account properly for the sampling design in analyzing survey data because the sampling design parameters (e.g., cluster size) have not been reported, and (3) the use of non-probability sampling designs.

Coverage bias results when the sampling frame excludes some members of the target population or includes ineligible persons who are not part of the target population. It can occur when certain districts of a country are excluded from a survey because of security considerations. It can also occur when members of the target population are homeless, nomadic, or institutionalized and therefore not identified with one of the physical homes used in the multistage household sampling designs common in low- and middle-income countries.

The household sampling methods designed by the World Health Organization for measuring child immunization coverage under the Expanded Programme on Immunization (EPI) provide a good example of the importance of considering sampling design when interpreting survey data. In this program, 30 clusters are selected and seven children within each cluster are selected for assessment of their immunization status [20],[21]. Variations of this protocol are widely used to measure sub-national intervention coverage in low- and middle-income countries as they require considerably fewer resources to implement than the well-established protocols employed in DHS and MICS surveys [8],[22], which enumerate all eligible households in the selected cluster and then make random selections from that updated list. While improvements have been made over the years, the EPI survey method does not typically include the development of an accurate sampling frame within each of the selected 30 clusters for selecting the seven children, and thus the survey design does not ensure that each child sampled has a known non-zero probability of selection [23]. Moreover, sampling weights cannot be used when analyzing these data to account for unequal probabilities of child selection, which requires accurate counts of the households in each cluster. As a result, selection bias cannot be ruled out, and may actually be quite likely when using traditional EPI 30×7 cluster survey methods [24]. Box 2 presents a hypothetical example of these issues.

**Box 2. Selection Bias Resulting from a Two-Stage Cluster Survey Protocol Where Complete Enumeration of Households within Primary Sampling Units Is Not Undertaken (Original EPI Method)**

Rapid surveys to assess sub-national intervention coverage in low- and middle-income countries often follow a variation of the “30×7 EPI” protocol where 30 villages are selected at random from a list of villages within a given area, typically with the probability of selection based on the estimated relative number of households in each villages provided by local officials or census data (probability proportional to size sampling). Within each village (cluster), the survey team selects seven households for interview to ascertain information on intervention coverage, most typically immunization coverage [8]. The procedure used for the selection of households in each cluster often involves beginning at a central area of the selected village, counting all households in a randomly selected direction from the center of the village to the village edge (a.k.a., “spin the bottle technique”), selecting a starting household randomly and then proceeding always to the next closest house until seven children aged 12–23 months have been found [20],[22]. This protocol can be more prone to selection bias than the household enumeration protocol used by DHS and MICS surveys for several reasons.

- If all households within selected villages are not enumerated to create an accurate sampling frame from which the seven households are selected at the second stage, one cannot document that each household in the village has a known and non-zero probability of selection, which is a requirement for probability sampling.
- It is rare that the true number of households in a selected village is equal to the estimated numbers used when selecting the 30 villages. This is not an issue if a true count of households in each of the 30 villages is obtained after selection, which then allows the survey data to be weighted during analysis to adjust for discrepancies in estimated and actual village sizes. However, if an accurate count of households is not obtained, the survey data cannot be weighted, and selection bias due to unequal probability of household selection cannot be ruled out.
- There is no requirement to document potentially eligible individuals in each household and conduct revisits. Any household where respondents are absent at the time of the visit is simply replaced by the nearest household having an eligible individual and respondent.

MNCH parameters are also sometimes assessed using “rapid” survey protocols that are designed to use minimal samples sizes to obtain local estimates of intervention coverage in comparison with a desired value for programmatic purposes. The small sample size survey technique called Lot Quality Assurance Sampling (LQAS) is a good example [25]. There are several varieties of this survey technique with varying levels of rigor [26]–[31], all of which seek to classify districts as having either adequate or inadequate intervention coverage. LQAS surveys are usually short, consisting of a few questions focused on a small number of binary outcomes. They are quick to administer and analyze, sometimes taking only 1–2 days per district. When implemented using best practices, study teams select households randomly from an up-to-date sampling frame so the results are representative of the population. At other times, however, respondents are identified using a convenience sample approach in which only one person is selected in a truly random fashion and subsequent respondents are selected from nearby households. Additionally, in some LQAS protocols field staff stop collecting data when it becomes clear that the final few respondents will not affect the district's adequate/inadequate classification. In all cases where an LQAS design does not allow each respondent to have a known and non-zero probability of selection, the sampling is not truly random and the validity of results is threatened by selection bias.

Non-response bias is also a common source of selection bias. Non-response bias results when the answers to survey questions differ between selected respondents who participate in the survey and selected respondents who choose not to participate, or are unable to participate, in the survey. The response rate is measured as the proportion of individuals selected to take part in a survey who actually complete the questionnaire. The potential for non-response bias goes up as the response rate goes down. This type of bias is common in phone and internet-based surveys—interview methods more commonly used in high-income countries where populations have access to such technology. In such surveys, it is not uncommon to have response rates as low as 50%, which must be corrected for by using survey weighting and other techniques. In low- and middle-income countries where survey interviews are conducted in person with individuals in selected households, non-response bias is usually less of an issue. The protocol used by DHS, MICS, AIS, and MIS surveys for limiting non-response bias requires interviewers to return to selected households at least three times if selected individuals in the household are unavailable for the interview [1]. This practice greatly limits household and individual non-response, with response rates among eligible women of reproductive age in these surveys typically exceeding 95%. However, non-response bias can be a serious issue for estimates of MNCH intervention coverage obtained from surveys that do not follow this protocol. For example, EPI surveys do not require multiple visits to selected households and do not document how many potentially eligible households were skipped because the respondent was absent. [23]. Both DHS and MICS surveys report the response rates of households and of eligible women and men who were selected to participate in the survey. This practice should be followed in all survey reports, and where response rates are below 85%–90%, surveys results should be interpreted with caution.

## Recommendations

Our major recommendations for interpreting MNCH coverage estimates are summarized in Box 3, but here we will briefly draw together the recommendations we make in this review.

**Box 3. Recommendations for Interpreting MNCH Coverage Indicators Measured from Household Surveys**

- Program managers and policy-makers must consider both sampling and non-sampling error when using MNCH coverage estimates for programmatic decision making and assessing progress against coverage targets.
- Study planners need to anticipate as many threats to the validity of the survey estimates as possible and put careful controls in place to limit the magnitude of non-sampling error.
- To help with interpreting MNCH intervention coverage estimates, additional validation research that identifies the sources, direction, and magnitude of non-sampling error of these estimates is needed.
- All survey reports that present MNCH intervention coverage estimates should provide detailed descriptions of the sampling design, sample size, survey instruments, quality controls, data analysis, and data collection protocols to improve the transparency, consistency, and interpretability of estimates.
- To allow for the consideration of important sources of non-sampling error in interpreting MNCH coverage estimates, survey reports should include a detailed limitations section that explicitly lists possible sources of non-sampling error, and should speculate about their direction and magnitude where possible.

MNCH intervention coverage estimates should be interpreted taking into account the confidence interval around the estimates, especially when assessing trends over time in intervention coverage, or comparing coverage across population subgroups or between geographic regions. To allow this to occur, we recommend that confidence intervals around key health indicators are included in the tables in the main body of all survey reports (not just in the appendices of DHS and MICS reports as at present). MNCH intervention coverage estimates with a high degree of uncertainty (e.g., because they are based on small subpopulations) should be interpreted with caution. As surveys are designed to provide valid intervention coverage point estimates only for survey domains or at higher levels, coverage estimates below the survey domain level should also be interpreted with extreme caution (e.g., coverage estimates at the district level when the survey was designed to yield valid estimates only at higher administrative levels).

To limit information error and bias, we recommend that extensive pretesting is conducted prior to survey implementation. Where possible, the results of pretesting should be used to adapt survey questionnaires to local norms and cultures, while safeguarding the key structure of the questions needed to ascertain standardized MNCH indicators in a consistent manner across countries and over time.

To limit selection bias, we recommend that MNCH estimates are obtained using well-established multi-stage sampling protocols, such as those used by DHS and MICS surveys, which create accurate sampling frames of households for second stage selection, weight data for unequal probability of selection, and return to households at least three times to limit non-response bias.

Importantly, we commend recent calls for establishing reporting guidelines for survey research [32]. We recommend that all survey reports and papers presenting MNCH intervention coverage estimates follow similar recommendations to improve transparency, consistency, and interpretability of estimates, even if detailed methods have to be included as a web appendix (see Box 3).

Finally, to allow for the consideration of important sources of non-sampling error in interpreting MNCH coverage estimates, survey reports should include a limitations section that explicitly lists possible sources of non-sampling error (see Box 3). Descriptive statistics on data quality that impact non-sampling error should be considered when interpreting MNCH coverage estimates, including household, individual and question non-response, socio-demographic composition of respondents, completeness of reporting, and date and age heaping. The DHS provides such information in an appendix to their country reports; other survey reports should do so as well.

## Conclusions

Estimates of MNCH intervention coverage obtained from household surveys are increasingly relied upon to assess progress in program effectiveness, and are a key metric in assessing Millennium Development Goals 4 and 5 for reducing neonatal, child, and maternal deaths worldwide. Surveys are the best tool we have for measuring MNCH intervention coverage at the population level and are central to national and global decision making but are subject to both sampling and non-sampling error. Non-sampling error is more insidious than sampling error and is difficult to quantify. It can, therefore, render the interpretation of MNCH intervention coverage estimates challenging. To this end, validation research that identifies the sources, direction, and magnitude of non-sampling error of MNCH intervention coverage estimates is urgently needed. Research presented elsewhere in this collection provides an excellent start [8]–[13], but future research must focus on refining and improving survey-based coverage estimates, and on developing a better understanding of how results on MNCH intervention coverage should be reported and interpreted.

**Key Points**

- Household surveys are the best tool we currently have for measuring MNCH intervention coverage at the population level in low- and middle-income countries and are central to national and global decision making.
- All estimates of MNCH intervention coverage obtained from household surveys have a certain level of error (total survey error) comprising sampling and non-sampling error, both of which must be considered when interpreting survey results.
- Sampling error (the precision of a point estimate) is measurable and is represented by the 95% confidence intervals, which characterize the precision of the estimate and provide context about whether estimated parameters are likely to be equal between two populations or time-points.
- Information error and bias are common sources of non-sampling error in household survey estimates; survey methods should be reported in enough detail to allow assessment of the potential for non-sampling error, the direction and magnitude of which is almost always unmeasurable.
- The focus of future research for measuring MNCH intervention coverage should be on refining and improving survey-based coverage estimates and developing a better understanding of how results should be interpreted and used.

## Abbreviations

AIS: AIDS Indicator Survey/s
DHS: Demographic and Health Survey/s
EPI: Expanded Programme on Immunization
ITN: insecticide-treated mosquito net
LQAS: Lot Quality Assurance Sampling
MICS: Multiple Indicator Cluster Survey/s
MIS: Malaria Indicator Survey/s
MNCH: maternal, newborn, and child health

## References

[1] Hancioglu A, Arnold F (2013) Measuring coverage in MNCH: Tracking progress in health for women and children using DHS and MICS household surveys. PLoS Med 10: e1001391 doi:10.1371/journal.pmed.1001391. 23667333 10.1371/journal.pmed.1001391 PMC3646216

[2] Groves RM, Fowler FJ, Couper MP, Lepkowski JM, Singer E, et al.. (2009) Survey methodology. Hoboken (New Jersey): John Wiley and Sons.

[3] Levy PS, Lemenshow S (1999) Sampling of populations: methods and applications. New York: John Wiley and Sons Inc.

[4] Payton ME, Greenstone MH, Schenker N (2003) Overlapping confidence intervals or standard error intervals: what do they mean in terms of statistical significance? J Insect Sci 3: 34. 15841249 10.1093/jis/3.1.34 PMC524673

[5] Schenker N, Gentleman JF (2001) On judging the significance of differences by examioning the overlap between confidence intervals. Am Stat 55: 182–186.

[6] International CSAEaI (2012) Ethiopia Demographic and Health Survey 2011. Addis Ababa, Ethiopia and Calverton (Maryland): Central Statistical Agency and ICF International.

[7] Central Statistics Authority (CSO), Ministry of Health (MOH), Tropical Disease Research Centre (TDRC), University of Zambia Macro International Inc. (2009) Zambia Demographic and Health Survey 2007. Calverton (Maryland): CSO and Macro International Inc.

[8] Cutts FT, Izurieta H, Rhoda D (2013) Measuring coverage in MNCH: Design, implementation, and interpretation challenges associated with tracking vaccination coverage using household surveys. PLoS Med 10: e1001404 doi:10.1371/journal.pmed.1001404. 23667334 10.1371/journal.pmed.1001404 PMC3646208

[9] Fischer Walker CL, Fontaine O, Black RE (2013) Measuring coverage in MNCH: Current indicators for measuring coverage of diarrhea treatment interventions and opportunities for improvement. PLoS Med 10: e1001385 doi:0.1371/journal.pmed.1001385. 23667330 10.1371/journal.pmed.1001385 PMC3646204

[10] Campbell H, el Arifeen S, Hazir T, O'Kelly J, Bryce J, et al (2013) Measuring coverage in MNCH: Challenges in monitoring the proportion of young children with pneumonia who receive antibiotic treatment. PLOS Med 10: e1001421 doi:10.1371/journal.pmed.1001421. 23667338 10.1371/journal.pmed.1001421 PMC3646212

[11] Eisele TP, Silumbe K, Yukich J, Hamainza B, Keating J, et al (2013) Measuring coverage in maternal and child health: Accuracy of measuring diagnosis and treatment of childhood malaria from household surveys in Zambia. PLoS Med 10: e1001417 doi:10.1371/journal.pmed.1001417. 23667337 10.1371/journal.pmed.1001417 PMC3646207

[12] Hazir T, Bequem K, el Arifeen S, Khan AM, Huque MH, et al (2013) Measuring coverage in MNCH: A prospective validation study in Pakistan and Bangladesh on measuring correct treatment of childhood pneumonia. PLoS Med 10: e1001422 doi:10.1371/journal.pmed.1001422. 23667339 10.1371/journal.pmed.1001422 PMC3646205

[13] Stanton CK, Rawlins B, Drake M, dos Anjos M, Cantor D, et al (2013) Measuring coverage in MNCH: Testing the validity of women's self-report of key maternal and newborn health interventions during the peripartum period in Mozambique. PLoS ONE 8: e60694 doi:10.1371/journal.pone.0060694. 23667427 10.1371/journal.pone.0060694 PMC3646219

[14] Suarez L, Simpson DM, Smith DR (1997) Errors and correlates in parental recall of child immunizations: effects on vaccination coverage estimates. Pediatrics 99: E3. 10.1542/peds.99.5.e3 9113960

[15] Valadez JJ, Weld LH (1992) Maternal recall error of child vaccination status in a developing nation. Am J Public Health 82: 120–122. 1536315 10.2105/ajph.82.1.120 PMC1694427

[16] Rhodes AE, Lin E, Mustard CA (2002) Self-reported use of mental health services versus administrative records: should we care? Int J Methods Psychiatr Res 11: 125–133. 12459825 10.1002/mpr.130 PMC6878364

[17] RBM (2008) Guidelines for Core Population-based Indicators Calverton, Maryland: Roll Back Malaria, MEASURE *Evaluation*, World Health Organization, UNICEF. New York: UNICEF.

[18] Eisele TP, Macintyre K, Yukich J, Ghebremeskel T (2006) Interpreting household survey data intended to measure insecticide-treated bednet coverage: results from two surveys in Eritrea. Malar J 5: 36. 16677379 10.1186/1475-2875-5-36 PMC1501030

[19] Skarbinski J, Winston CA, Massaga JJ, Kachur SP, Rowe AK (2008) Assessing the validity of health facility-based data on insecticide-treated bednet possession and use: comparison of data collected via health facility and household surveys–Lindi region and Rufiji district, Tanzania, 2005. Trop Med Int Health 13: 396–405. 18397401 10.1111/j.1365-3156.2008.02014.x

[20] Lemeshow S, Robinson D (1985) Surveys to measure programme coverage and impact: a review of the methodology used by the expanded programme on immunization. World Health Statistics Quarterly 38: 65–75. 4002731

[21] Henderson RH, Sundaresan T (1982) Cluster sampling to assess immunization coverage: a review of experience with a simplified sampling method. Bull World Health Organ 60: 253–260. 6980735 PMC2535957

[22] Cromwell EA, Ngondi J, McFarland D, King JD, Emerson PM (2012) Methods for estimating population coverage of mass distribution programmes: a review of practices in relation to trachoma control. Trans R Soc Trop Med Hyg 106: 588–595. 22884927 10.1016/j.trstmh.2012.07.011

[23] Turner AG, Magnani RJ, Shuaib M (1996) A not quite as quick but much cleaner alternative to the Expanded Programme on Immunization (EPI) Cluster Survey design. Int J Epidemiol 25: 198–203. 8666490 10.1093/ije/25.1.198

[24] Luman ET, Worku A, Berhane Y, Martin R, Cairns L (2007) Comparison of two survey methodologies to assess vaccination coverage. Int J Epidemiol 36: 633–641. 17420165 10.1093/ije/dym025

[25] Robertson SE, Valadez JJ (2006) Global review of health care surveys using lot quality assurance sampling (LQAS), 1984–2004. Soc Sci Med 63: 1648–1660. 16764978 10.1016/j.socscimed.2006.04.011

[26] Rhoda DA, Fernandez SA, Fitch DJ, Lemeshow S (2010) LQAS: user beware. Int J Epidemiol 39: 60–68. 20139433 10.1093/ije/dyn366

[27] Pezzoli L, Tchio R, Dzossa AD, Ndjomo S, Takeu A, et al (2012) Clustered lot quality assurance sampling: a tool to monitor immunization coverage rapidly during a national yellow fever and polio vaccination campaign in Cameroon, May 2009. Epidemiol Infect 140: 14–26. 21418714 10.1017/S0950268811000331

[28] Pezzoli L, Andrews N, Ronveaux O (2010) Clustered lot quality assurance sampling to assess immunisation coverage: increasing rapidity and maintaining precision. Trop Med Int Health 15: 540–546. 20214765 10.1111/j.1365-3156.2010.02482.x

[29] Olives C, Pagano M (2010) Bayes-LQAS: classifying the prevalence of global acute malnutrition. Emerg Themes Epidemiol 7: 3. 20534159 10.1186/1742-7622-7-3 PMC2903572

[30] Hedt BL, Casey O, Pagano M, Valadez JL (2008) Large country-lot quality assurance sampling: a new method for rapid monitoring and evaluation of health, nutrition and population programs at sub-national levels. Washington (D.C.): The World Bank. p 1–61.

[31] Olives C, Valadez JJ, Brooker SJ, Pagano M (2012) Multiple category-lot quality assurance sampling: a new classification system with application to schistosomiasis control. PLoS Negl Trop Dis 6: e1806 doi:10.1371/journal.pntd.0001806. 22970333 10.1371/journal.pntd.0001806 PMC3435238

[32] Bennett C, Khangura S, Brehaut JC, Graham ID, Moher D, et al (2011) Reporting guidelines for survey research: an analysis of published guidance and reporting practices. PLoS Med 8: e1001069 doi:10.1371/journal.pmed.1001069. 10.1371/journal.pmed.1001069 PMC3149080 21829330

[33] Central Statistical Agency [Ethiopia] and ICF International (2012) Ethiopia Demographic and Health Survey 2011. Addis Ababa, Ethiopia and Calverton (Maryland): Central Statistical Agency and ICF International. Available: http://www.measuredhs.com/pubs/pdf/FR255/FR255.pdf. Accessed 11 December 2012.

